# Progress towards Patient-Specific, Spatially-Continuous Radiobiological Dose Prescription and Planning in Prostate Cancer IMRT: An Overview

**DOI:** 10.3390/cancers12040854

**Published:** 2020-04-01

**Authors:** Emily Jungmin Her, Annette Haworth, Pejman Rowshanfarzad, Martin A. Ebert

**Affiliations:** 1 Department of Physics, University of Western Australia, Crawley, WA 6009, Australia; 2 Institute of Medical Physics, University of Sydney, Camperdown, NSW 2050, Australia; 3 Department of Radiation Oncology, Sir Charles Gairdner Hospital, Nedlands, WA 6009, Australia; 4 5D Clinics, Claremont, WA 6010, Australia

**Keywords:** prostate cancer, precision medicine, biological optimisation

## Abstract

Advances in imaging have enabled the identification of prostate cancer foci with an initial application to focal dose escalation, with subvolumes created with image intensity thresholds. Through quantitative imaging techniques, correlations between image parameters and tumour characteristics have been identified. Mathematical functions are typically used to relate image parameters to prescription dose to improve the clinical relevance of the resulting dose distribution. However, these relationships have remained speculative or invalidated. In contrast, the use of radiobiological models during treatment planning optimisation, termed biological optimisation, has the advantage of directly considering the biological effect of the resulting dose distribution. This has led to an increased interest in the accurate derivation of radiobiological parameters from quantitative imaging to inform the models. This article reviews the progress in treatment planning using image-informed tumour biology, from focal dose escalation to the current trend of individualised biological treatment planning using image-derived radiobiological parameters, with the focus on prostate intensity-modulated radiotherapy (IMRT).

## 1. Introduction

It is well-established that prostate cancer (PCa) exhibits high multifocality and heterogeneity [1,2,3] and yet the current standard of care for PCa with external beam radiotherapy (EBRT) still largely remains a prescription of uniform, conformal dose distribution. Whole-gland therapy has traditionally been used due to the difficulty in precisely targeting tumour foci using conventional imaging techniques. With the advance in quantitative imaging, mainly magnetic resonance imaging (MRI) and positron emission tomography (PET), focal dose escalation to the dominant lesion has become popular.

“Dose painting” was first proposed by Ling et al. [4] to target tumour characteristics in addition to tumour location from quantitative imaging. Tumour subvolumes with a potential relative radiation resistance were identified and prescribed an escalation dose (a “boost dose”). This initial definition of dose painting is now labeled dose-painting-by-contours (DPbC) where the boost subvolume(s) within the target is created with image parameter threshold(s). The disadvantage with discrete volumes is that they are binary: Voxels inside a volume are assumed to represent a tumour or more aggressive disease. In reality, the tumour biology characteristics are continuous in a three-dimensional space. A more precise dose painting technique is called “dose-painting-by-numbers” (DPbN) [5]. The principle is to apply dose prescription at the voxel-level according to tumour characteristics such as clonogen density. Advanced imaging methods that can reveal biological information at the voxel level are now available, along with image-guided radiotherapy (IGRT), making DPbN feasible.

**Figure 1:**
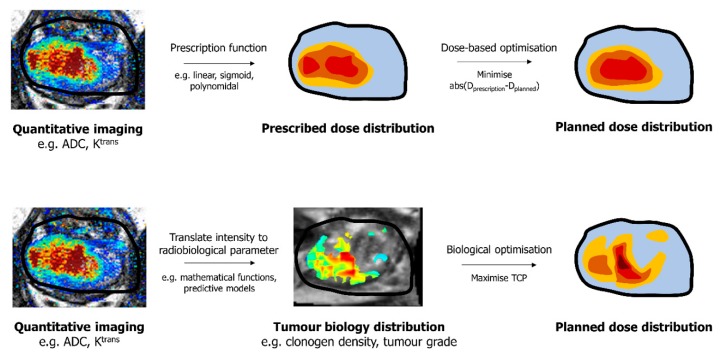
Pathways for optimising planned dose distribution based on the desired dose prescription (**top**) and based on the desired endpoint (**bottom**) using the same quantitative imaging data.

DPbN requires demonstration of correlation between imaging features and specific tumour biology characteristics [5,6,7] and a mathematical relationship to map an image-derived parameter to a voxel-level prescription dose. DPbN attempts to incorporate imaging data as a surrogate of biological information to improve clinical relevance. The top row of Figure 1 illustrates how quantitative imaging might be used to define an objective dose prescription for plan optimisation. However, conventional dose-based objectives rarely represent optimal tumour control. The ultimate goal of radiotherapy is to eradicate a tumour with minimal toxicity, which translates to a maximum tumour control probability (TCP) and minimum normal tissue complication probability (NTCP). Therefore, ideally, accurate radiobiological models of TCP and NTCP should be incorporated in both treatment planning and evaluation to produce dose distributions that are more likely to result in the desired treatment outcome (endpoint) rather than traditional dose-based planning objectives. A mechanistic TCP model requires the determination of voxel-level parameters, and the bottom row of Figure 1 illustrates how the same quantitative imaging might be used to obtain that information.

This article aims to review the progress in treatment planning using image-informed tumour biology, from focal dose escalation to the current trend of individualised biological treatment planning using voxel-level, image-derived radiobiological parameters to achieve optimal treatment outcomes, with the focus on prostate IMRT.

## 2. Image-Guided Focal Dose Escalation in PCa IMRT 

Follow-up studies on treating PCa with a uniform-dose distribution have shown that local recurrences typically occur at the site of the primary tumour [8,9,10,11], suggesting the insufficiency of the conventional prescription dose. Dose escalation to the entire prostate gland is not practical as improved tumour control would be achieved at the expense of unacceptable normal tissue toxicity. Instead, delivering a focal boost dose to the dominant intraprostatic lesion (DIL) while irradiating the rest of the gland with the conventional prescription dose became popular. Concerns surrounding focal dose escalation include difficulty in accurately delineating DIL(s) [12]. Evolution of PCa quantitative imaging, multiparametric MRI (mpMRI) in particular, has been the driving force behind focal intraprostatic dose escalation. mpMRI is the use of multiple anatomical and quantitative MR sequences to image different tumour characteristics and has demonstrated improved sensitivity and specificity in cancer detection compared to using anatomical T2-weighted imaging (T2W) alone [13]. 

The FLAME trial investigated the treatment efficacy of focal dose escalation, the largest and the first randomised trial of its kind [14]. The FLAME trial used mpMRI to delineate DIL. Intermediate- and high-risk PCa patients were randomised into the standard arm that delivered 77 Gy to the whole gland over 35 fractions or the experimental arm that received a focal boost to the DIL resulting in a total dose of 95 Gy. Focal dose escalation demonstrated comparable genitourinary (GU) and gastrointestinal (GI) toxicity to the standard arm up to two years post-treatment [15]. Murray et al. recently reported on preliminary results from the DELINEATE trial that applied focal dose escalation to conventionally-fractionated and hypofractionated IMRT for intermediate- and high-risk PCa [16]. An escalated dose of 82 Gy in 37 fractions (whole-gland dose 74 Gy/ 37 fractions) and 60 Gy to 20 fractions (whole-gland dose 67 Gy/20 fractions) was delivered to mpMRI-defined DIL. Similar normal tissue toxicity was observed in a one-year follow-up in comparison with studies without focal dose escalation. Results from other smaller studies involving focal dose escalation to mpMRI-defined DIL appear promising with good tumour control and favourable toxicity profiles [17,18]. 

## 3. Deriving the Desired Dose Prescription from Voxel-Level Information

A major limitation of escalating the dose in only the DIL delineated on imaging is the risk of missing other biologically significant tumours as PCa is a multifocal disease. Furthermore, a large inter-observer variation in DIL delineation on multimodality imaging has been observed [19,20,21,22,23]. While DPbC overcomes these shortcomings by systemically identifying subvolumes for homogeneous dose escalation, it fails to utilise the voxel-level tumour characteristic information provided by imaging in dose prescription. As DPbN has the potential to offer a more sophisticated method to deliver a spatially-varying dose distribution using the full range of image parameters, we will now focus on DPbN as the next step in incorporating image-informed tumour biology in treatment planning.

**Figure 2:**
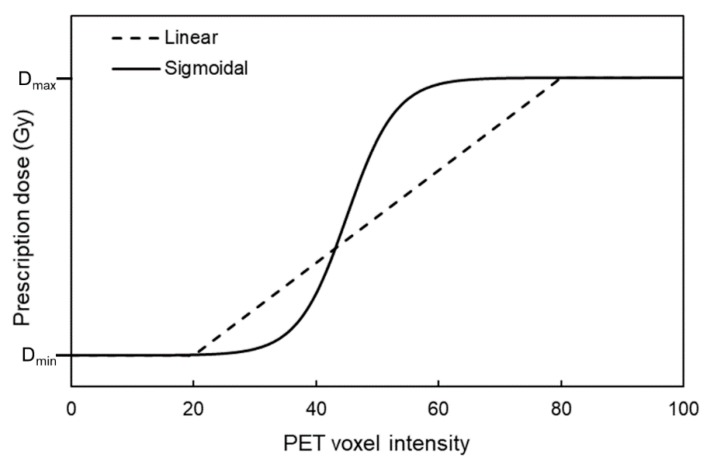
Linear and nonlinear (sigmoidal) prescription function relating the positron emission tomography (PET) voxel intensity to a local dose prescription for dose-painting-by-numbers (DPbN).

DPbN essentially establishes a mathematical link between imaging parameters and dose prescriptions that optimise chosen clinical endpoints [4,5]. The majority of DPbN studies assumed a linear relationship between image intensity and the required boost dose [24,25,26,27,28]. The linear function usually extends from a minimum dose, typically set to the current clinical dose prescription, and a maximum dose, set to a value that is considered “safe” for the target (Figure 2). The linear prescription function that is most widely adopted comes from the work of Vanderstraeten et al. [28]:

(1) $$D_{i}=D_{low}\;\;\;\;\;\;\;\;\;\;\;\;\;\;\;\;\;\;\;\;\;\;\;\;\;\;\;\;\;\;\;\;\;\;\;\;\;\;\;\;\;\;\;\;if\;I_{i}\leq I_{low}$$

(2) $$D_{i}=D_{low}+\frac{I_{i}-I_{low}}{I_{high}-I_{low}}\;\left(D_{high}-D_{low}\right)\;if\;I_{low}<I_{I}<I_{high}$$

(3) $$D_{i}=D_{high}\;\;\;\;\;\;\;\;\;\;\;\;\;\;\;\;\;\;\;\;\;\;\;\;\;\;\;\;\;\;\;\;\;\;\;\;\;\;\;\;\;\;if\;I_{i}\geq I_{high}$$

where dose values increase linearly with voxel intensity ($I_{i}$) between $D_{low}$ and $D_{high}$, respectively, corresponding to $I_{low}$ and $I_{high}$. In this work, *I_low_* was not chosen as the lowest intensity found in the image to minimise the effect of noise. Instead, the dose was escalated between 25% and 100% of the 95th percentile PET voxel intensity values. 

**Figure 3:**
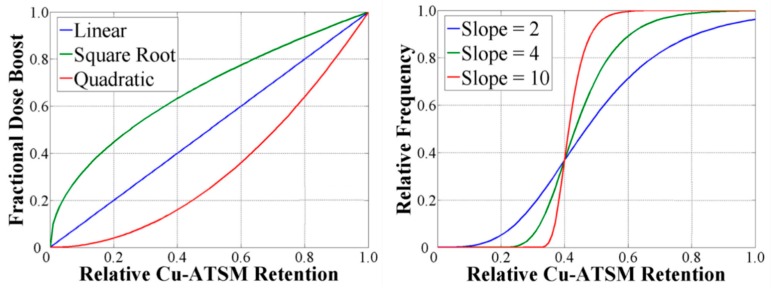
Quantitative forms for prescription functions. Left: Polynomial prescriptions, and right: Sigmoid prescriptions [29]. ©Institute of Physics and Engineering in Medicine. Reproduced by permission of IOP Publishing. All rights reserved.

Other commonly used prescription functions include sigmoidal (Figure 2) and polynomial functions. Sigmoidal functions capture the biological and clinical effects of local dose deposition. As there is no consensus on the optimal dose prescription, Bowen et al. investigated the sensitivity of IMRT planned dose conformity to variations in the mathematical function used [29]. The planning target volume (PTV) dose was set to 70 Gy plus a hypoxia surrogate uptake-dependent boost dose. Cu61-ATSM PET was used as the surrogate for cellular hypoxia. Two prescription functions were used for surrogate uptake dependence, in the form of either an *n*th order polynomial function or a sigmoidal function (Figure 3). Prescription functions based on polynomials were found to be the least constraining on their optimised plans, while prescriptions based on a sigmoid mapping function were the most demanding to deliver. Interestingly, integral doses to normal tissues were insensitive to the shape of the prescription function. This result demonstrates that as long as normal tissue constraints are met, treatment efficacy strongly depends on the ability to achieve the required dose dictated by tumour characteristic heterogeneities. 

Instead of image intensities, quantitative parameters derived from imaging can also be used in the prescription function for improved biological relevance. One example is from the work of van Schie et al. [30] where a tumour probability (TP) map was used in a polynomial dose prescription function. The prescription dose was computed by:

(4) $$D_{presc,i}=D_{min}+\left(D_{max}-D_{min}\right)\times TP_{i}^{n}$$

where $D_{presc,i}$ is the prescription dose for voxel i. $D_{min}$ and $D_{max}$ are the minimum and maximum prescribed dose, respectively, and n is the polynomial order of the mapping function. In their study, voxel-level TP maps were derived from 30 image features from mpMRI using a logistic regression model in combination with a priori tumour location information from radical prostatectomy patients. 

A major drawback of DPbN studies is the use of arbitrarily-chosen functions that have not been validated against clinical outcomes data [24,25,29,31,32,33,34]. They are likely to be an oversimplification of the complex tumour dose-response. Furthermore, the maximum dose allowed in the focal dose escalation studies is chosen such that it has previously demonstrated safety [14,35,36], but it does not necessarily ensure optimal tumour control. These, together with the limitation of dose-based treatment planning in producing patient-specific plans, make biological optimisation attractive. 

## 4. Deriving a Dose Distribution with the Desired Endpoint from Voxel-Level Information

Biological optimisation could be broadly interpreted as any treatment planning method that incorporates biological information, but for this review, it is defined as an optimisation technique that uses a TCP model as the target objective. TCP was chosen as the focus as it directly represents the desired treatment outcome.

Early formulations of the modern linear-quadratic (LQ) model appeared in the late 1940s [37,38] and became more established with extensive investigations in the 1970s and 1980s [39,40,41,42,43,44,45,46,47,48,49]. It has now become a preferred simplified mathematical description of cell survival in response to radiation. The model assumes that there are two components to the radiation damage; the linear component, *α*, where the number of cell deaths is proportional to the dose; and the quadratic component, *β*, where the number of cell deaths is proportional to the square of the dose. α represents a single photon causing a lethal damage (intrinsic radiosensitivity of the tumour). β represents two or more photons each causing a sublethal (potentially-repairable) damage, combining into a lethal form of damage (repair capability of the tumour). The survival fraction (SF) can be expressed as:

(5) $$SF=\exp\left(-\alpha D-\beta D^{2}\right)$$

where D is the total dose.

If it is assumed that any surviving clonogenic cell may repopulate a tumour, then the probability of tumour control is the same as the probability that all clonogenic cells are killed, given by:

(6) $$TCP=\exp\left(-N_{initial}\times SF\right)$$

where $N_{initial}$ is the initial number of clonogenic cells and SF is the surviving fraction after a course of treatment. 

For fractionated EBRT with n  fractions and d fractional dose in voxel i, the TCP of the corresponding voxel is formulated as:

(7) $$TCP_{i}=\exp\left(-\rho_{i}V_{i}exp\left(-\alpha nd_{i}-\beta nd_{i}^{2}\right)\right)$$

The initial number of clonogenic cells in voxel i, $N_{initial,i}$ can be expressed as the product of clonogen density per volume, $\rho_{i}$, and volume of the voxel, $V_{i}$.

Then, the overall TCP of a given dose distribution within the target structure is calculated as the product of all voxel TCPs:

(8) $$TCP=\overset{N}{\underset{i}{\prod }}TCP_{i}$$

where N is the total number of voxels in the target structure. The formulation requires an assumption that individual subregions of the tumour respond independently to the response of other subregions or nonlocal dose effects. 

Taking account of the tumour growth dynamics where the combined influence of cell doubling, the proportion of proliferating cells, and accelerated clonogen proliferation are considered, the TCP can then be described by:

(9) $$TCP_{i}=\exp\left(-\rho_{i}V_{i}exp\left(-\alpha nd_{i}-\beta nd_{i}^{2}+ln\left(2\right)\frac{T_{exp}-T_{k}\;}{T_{pot}}\right)\right)$$

Here, $T_{exp}$ is the overall treatment time, $T_{k}$ is the kick-off time of the accelerated repopulation, and $T_{pot}$ is the potential doubling time. In applications to EBRT, temporal dependence on sublethal damage repair tends to be ignored but can be included for protracted treatments [50]. 

As radiosensitivity varies among individuals in a population, inter-patient radiosensitivity can be taken into account by applying a population distribution to the radiosensitivity parameter *α* and then utilising a normalisation with a weighted sum:

(10) $$TCP_{i}=\frac{\sum_{k}w\left(\alpha_{k}\right)TCP_{i}\left(\alpha_{k}\right)}{\sum_{k}w\left(\alpha_{k}\right)}$$

where $w\left(\alpha_{k}\right)$ is the population distribution function of α. A common quantitative form is that of a normal distribution, described by a mean $\bar{\alpha }$ and standard deviation $\sigma_{\alpha }$. 

Hypoxic tumour cells are radioresistant and hence the presence of hypoxia alters the radiosensitivity parameters. The amount which the tumour radiosensitivity changes is described by the oxygen enhancement ratio (OER):

(11) $$\alpha_{hypoxic}=\frac{\alpha }{OER}$$

(12) $$\beta_{hypoxic}=\frac{\beta }{OER^{2}}$$

## 5. Biological Optimisation of Prostate IMRT Using Population-Based Parameters

Early attempts in incorporating TCP in biological optimisation often involved maximisation of subvolume TCP with limited modeling of intra-tumour heterogeneity from quantitative imaging. Kim and Tomé [51] used a phenomenological TCP model that only has two radiobiological parameters, the dose which yields a TCP of 50%, $D_{50}$, and the slope of the TCP curve at $D_{50}$, $\gamma_{50}$:

(13) $$TCP\left(D\right)=\frac{1}{1+{\left(\frac{D_{50}}{D}\right)}^{4\gamma_{50}}}$$

**Figure 4:**
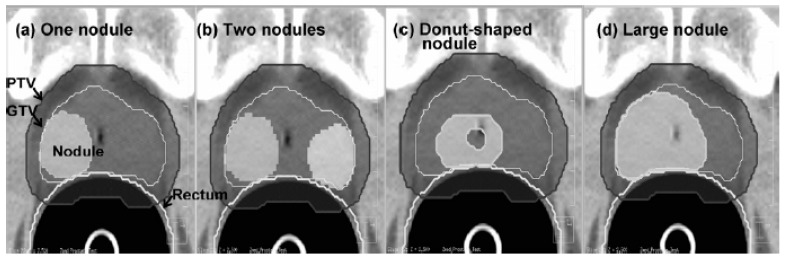
Subvolume (nodule) geometries. (**a**) One-nodule case, (**b**) two-nodule-case, (**c**) donut-shaped-nodule case, (**d**) big-nodule case. Reproduced with permission from [51].

Each subvolume was assigned different values of the radiobiological parameters depending on the risk of recurrence. All voxels within a subvolume were assumed to possess identical tumour characteristics. $D_{50}$ values were derived from the published clinical data of Levegrün et al. [52]. A variety of subvolume (nodule) geometries was created arbitrarily to test the feasibility of biological optimisation (Figure 4). The biological optimisation aimed to maximise TCP while simultaneously minimising NTCP [53]. For all subvolume geometries investigated, the biological optimisation was feasible without a significant increase in NTCP compared to conventional uniform-dose plans. 

Uzan et al. [54] maximised the TCP of the DIL, identified on quantitative MRI, while satisfying the NTCP limits for grade 2–3 rectal bleeding and grade 3 faecal incontinence. For TCP modeling, it was assumed that all clonogens are located uniformly inside the DIL with a constant clonogen density and any clonogens outside the DIL can be controlled by the dose to the clinical target volume (CTV). The radiosensitivity parameter, α, was assumed to be normally distributed within a population described by $\bar{\alpha }$ = 0.185 Gy^−1^ and $\sigma_{\alpha }$= 0.053 Gy^−1^, introducing inter-tumour heterogeneity. Biological optimisation was performed using research software and the resulting plan was replanned on clinical software using the dose-volume (DV) metrics. Uzan et al. reported the potential of biological optimisation in yielding high tumour control with promising toxicity profile. Only two patients experienced grade 2 urinary toxicity and two patients had ≤ grade 2 late rectal toxicity over a median follow-up of 36 months.

More sophisticated intra-tumour biology was modeled for Betts et al. and Haworth et al. [55,56,57], where they used location-dependent cell density distributions to drive biological optimisation of prostate low-dose-rate brachytherapy. The prostate was divided into the apex, mid, and base segments where each was further divided into four quadrants. The probability of finding a tumour in each quadrant [58] was used to assign an initial clonogen number to the corresponding quadrant. Biological optimisation determined the seed placement so that the overall TCP was maximised while maintaining the organs at risk (OAR) DV constraints and restrictions on seed arrangement. The biologically-optimised plans were able to reduce the dose to the urethra while maintaining a high TCP. This study also demonstrated robustness to random seed displacement compared to conventional plans.

## 6. Biological Optimisation of Prostate IMRT Using Patient-Specific, Voxel-Level Parameters

While population-based parameters can be extracted from large trial datasets, spatial voxel-level tumour characteristics are ideal for a patient-specific DPbN plan. Deriving an accurate, quantitative relationship between image features and radiobiological parameters is challenging, leading to very few treatment planning studies incorporating image-derived PCa parameters so far. 

Dircherl et al. [59] used prostate 18F-Choline PET images to derive a voxel-level proliferation rate and cell density which were then used to compute dose distributions with maximum TCP. An independent linear relationship between each parameter and standard uptake value from PET was assumed. A voxel-wise prescription function was derived such that it ensures a maximum TCP is given for a fixed target integral dose. While a voxel-level TCP was included in treatment planning, the derived plans were not truly “biologically-optimised” since the optimiser did not iteratively evaluate TCP to derive the optimal deliverable dose distribution that maximises TCP. Instead, an objective function that minimised the difference between the prescribed dose and the planned dose was used. While this approach is likely to produce a high TCP, the mathematical formulation of TCP dictates that maximal TCP is achieved when individual voxel TCPs are very high and identical to each other [60]. Therefore, the presence of one single voxel with a low TCP will limit the overall TCP. Failure to incorporate TCP evaluation within the optimiser could result in suboptimal treatment plans. 

Grönlund et al. [61] used a phenomenological TCP model in biological optimisation of prostate radiotherapy. Apparent diffusion coefficient (ADC) calculated from diffusion-weighted MR has been correlated with Gleason score as increased cellularity restricts diffusion [62,63]. Using published correlations, Gleason score of the voxel of interest, $G_{i}$, was determined from ADC map. Then, voxel TCP was computed as:

(14) $$TCP_{i}=\frac{1}{1+{\left(\frac{D_{50}\left(G_{i}\right)}{D}\right)}^{4\gamma_{50}\left(G_{i}\right)}}$$

where dose-response parameters $D_{50}$ and $\gamma_{50}$ are functions of the assigned Gleason score. Gleason score and dose-response were related by using preradiotherapy biopsy and postradiotherapy outcomes data from 122 patients treated with a uniform dose distribution. The parameters were derived such that they agreed with the observed freedom from biochemical failure rate at five years postradiotherapy. The proposed formalism demonstrated a potential for increasing TCP without extra dose burden. However, it was a dose redistribution study without the consideration of normal tissue toxicity. 

While improved radiobiological models have been used in biological optimisation, the derivation of the model parameters from imaging has largely been limited to invalidated functions of image intensity. The combination of radiomics and machine learning methods appears to be a potential solution for accurate image-derived radiobiological parameters. Radiomics aims to extract quantitative features from images. These features can then be used to develop predictive models using machine learning methods when “ground-truth” voxel-level data is available. Many investigators have explored and yielded promising results from using radiomics and machine learning of mpMRI for PCa detection [64,65,66,67,68], tumour aggressiveness (Gleason score) classification, and staging [62,69,70,71,72,73,74]. Voxel-level tumour biology distributions generated by predictive models using mpMRI can then be used to drive biological optimisation of prostate radiotherapy as demonstrated by the bottom row of Figure 1. 

**Figure 5:**
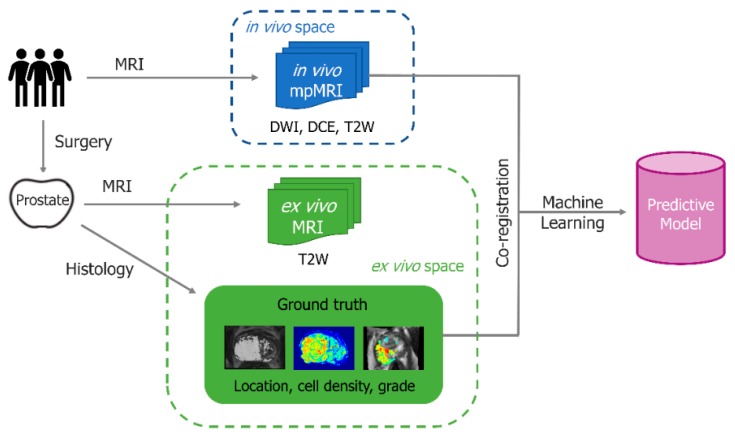
A diagram showing the predictive model development process for biofocused radiotherapy (BiRT). Prior to radical prostatectomy, in vivo multiparametric magnetic resonance imaging (mpMRI) data is collected. mpMRI feature extraction and selection is performed. Ground truth tumour biology maps are derived from removed prostate histology. Ground truth maps and mpMRI data are spatially registered using ex vivo and in vivo T2W images and machine learning methods are used to build predictive models. Regarding Figure 1, the resulting predictive models can be utilised to derive the tumour biology distribution from an individual patient’s multi-parametric images.

One such approach proposed by Haworth et al. is called the biofocused radiotherapy (BiRT) [57,75]. The BiRT approach utilises machine learning methods to generate voxel maps of tumour biology information from imaging data, which is then used in the calculation of a parameterised voxel-level TCP objective for plan optimisation. Using “ground-truth” histology information from a database of prostatectomy patients and presurgical mpMRI spatially registered with histology sections, reliable predictive models can be made without the explicit understanding of underlying biological and physical processes (Figure 5). Imaging biomarkers for predicting prostate tumour location, cell density, and tumour grade from mpMRI have been developed [65,71,76]. Image registration methods have recently been refined further to allow voxel-wise correlation of PET/CT with mpMRI and histology data of the prostate [77]. Work is currently underway to use the voxel-level, patient-specific tumour information from mpMRI and imaging biomarkers to drive biological optimization of prostate IMRT. Preliminary investigation has demonstrated a reduction in dose to the rectum and bladder for biofocused prostate IMRT compared to conventional dose- and DV-based methods [78,79]. 

## 7. Ongoing and Future Considerations

The future of prostate radiotherapy treatment planning is promising with work underway to improve various aspects of biological optimisation. However, it is still far from clinical deployment, largely owing to uncertainties in radiobiological models and their parameters. In addition to methods of incorporating these uncertainties in treatment planning optimisation, hypofractionation and adaptive therapy are of interest in PCa radiotherapy. It is expected that future research addressing these issues, some of which are outlined below, will lead to the realisation of patient-specific, biologically-optimised radiotherapy as the standard of care.

### 7.1. More Complete TCP Models

There are several significant limitations in current TCP models that need to be overcome. Firstly, these models are usually mechanistic in nature and lack the ability to incorporate important nondosimetric clinical factors such as age, interactions with other treatment responses. Secondly, the TCP model described above fundamentally assumes that clonogens do not communicate with each other, that cell killing events are independent and that clonogens only respond to local dose deposition. These assumptions ignore the bystander effect where cytotoxic effects are seen in nonirradiated cells. The indirect effects of ionising radiation in tumour control is another aspect of tumour biology that has not been incorporated in TCP modeling. One such effect is the vascular effect where high local doses create an unfavourable tumour microenvironment through vascular damage. In studies with experimental tumours, a dose/fraction size greater than 10 Gy has demonstrated the ability to induce severe vascular damage such as endothelial apoptosis that leads to tumour cell killing through the reduced blood supply, thus starving the tumour of oxygen and other nutrients [80,81,82,83]. It has also been suggested that high dose/fraction irradiation triggers an immune response and thereby eradicates tumour cells that escaped radiation-induced death [84]. The radiation-induced immune response is also thought to be related to the regression of lesions outside the radiation field, called the abscopal effect [85]. The above mechanisms, together with patient-specific genomic factors, can be integrated into a mechanistic radiobiological model as presented above. Such a model would, however, have a very large number of patient-specific, spatially and temporally-varying parameters. A considerable effort will be required to develop imaging or other techniques to derive such parameters. Sensitivity analyses can identify which parameters need to be derived with the highest priority [86].

### 7.2. Sensitivity and Specificity of Quantitative Imaging

The reporting of sensitivity and specificity of quantitative imaging in detecting tumour biology information is necessary for a complete understanding of the accuracy of a biological optimization approach in treatment planning. Prostate mpMRI has primarily been investigated in tumour detection and localisation demonstrating high sensitivity and specificity [87,88]. There is limited literature on other tumour characteristics such as cellular hypoxia as they largely remain in the investigational stage [89]. The effect of quantitative imaging uncertainties on treatment planning has also been investigated in a limited number of studies. For example, Kim and Tomé investigated the effect of imaging accuracy on biologically-optimised prostate IMRT plans [90]. Loss of sensitivity was modeled as misclassification of “high-risk” tumour voxels as “low-risk”; and loss in specificity as misclassification of “low-risk” tumour voxels as “high-risk”. Their results suggest that TCP of a biologically-optimised plan is more sensitive to a loss in quantitative imaging sensitivity compared to the loss in specificity. This is expected since a “low-risk” voxel classified as “high-risk” will still be treated with a sufficient dose whereas the optimiser cannot adequately account for a “high-risk” voxel misclassified as “low-risk”. 

For biological optimisation where a radiobiological model is informed by imaging via predictive models, quantitative imaging accuracy is combined with other model uncertainties as overall predictive model accuracy. Factors that influence model accuracy include reconstruction methods, machine learning methods, and size of the training dataset. The effect of the overall uncertainty in the predicted parameter value on the resultant plan should be performed on a case-by-case basis.

### 7.3. Robustness to Uncertainties

The standard approach to incorporate treatment delivery uncertainty in radiotherapy has been the use of a margin surrounding the CTV to generate a PTV [91]. It is assumed that the CTV only moves within the boundaries of the PTV and therefore ensures that the prescribed uniform dose is delivered to the CTV with a specific likelihood. However, the PTV concept has several limitations as discussed by Unkelbach et al. [92], which includes the inability to guarantee the optimal balance between tumour control and normal tissue sparing. Robust optimisation or probabilistic treatment planning methods have been developed to overcome PTV limitations by directly incorporating uncertainties in treatment planning optimisation [93,94,95,96,97,98,99,100]. Probabilistic treatment planning methods are particularly important for biological optimisation since the use of margins for nonuniform dose distributions within the CTV is problematic. Furthermore, it is relatively easier to incorporate uncertainties from the additional steps that involve imaging data, image registration, and radiobiological modeling. Clinical implementation of biological optimisation, therefore, requires knowledge of the sources and effects of geometric uncertainties on the dose distribution. An underestimation of geometric errors, the systematic component, in particular, may result in treatment failure. 

### 7.4. Hypofractionation

With a consensus that the α/β  ratio of PCa is less than 5 Gy [101,102,103] and the neighbouring rectum and bladder have a higher α/β ratio of 5-8 Gy [104,105], hypofractionation can be expected to improve treatment outcome. Many randomised clinical trials investigating treatment safety and efficacy of hypofractionated treatment compared to standard fractionation have been completed with encouraging results [106,107,108,109,110,111,112,113]. However, an increase in acute GI and late GU has been reported for hypofractionation studies [114,115] due to difficulty in maintaining OAR doses at acceptable levels while delivering a high uniform dose to the PTV. Biological optimisation has the potential to allow a reduction in dose to the OARs through more efficient distribution of dose to the PTV. Biological optimisation with population-based TCP and NTCP objective functions in the hypofractionated treatment of high-risk PCa has previously demonstrated good biochemical control with comparable urinary and bowel toxicity to conventional fractionation [116]. As such, the radiobiological advantage of hypofractionation may be better realised with patient-specific TCP models.

### 7.5. Adaptive Therapy

Standard radiotherapy assumes that the tumour response will be consistent throughout treatment. However, cancer is known to adapt to therapies and its biological characteristics are often temporally and spatially heterogeneous. To address the time-dependent changes in tumour characteristics, an adaptive therapeutic strategy has been proposed [117]. Adaptive therapy evaluates treatment response by comparing imaging data acquired before treatment commences (baseline) and at various time points during treatment to adapt the initial dose distribution accordingly. Hypoxia is one of the tumour characteristics often targeted for adaptive therapy due to its highly spatially- and temporally-variable nature and has primarily been investigated in head and neck cancer using PET with promising results [33,118,119,120,121]. There are, however, challenges to overcome before the clinical application of adaptive therapy can be realised. Adaptive therapy is labour-intensive, especially for biological optimisation since quantitative image acquisition and registration, analysis, and treatment planning must be completed for each stage of the therapy. While this may be impractical for conventional fractionation schedules, it is potentially feasible for PCa since the low *α/β* ratio allows extreme hypofractionation with less than five fractions. The optimal imaging frequency and timing remains unknown. 

## 8. Conclusions

Advances in quantitative imaging of PCa allow better targeting of the tumour using radiobiological information derived from the images. Clinical studies that focused on translating image parameters to the desired dose demonstrated promising results in improving tumour control with minimal normal tissue toxicity. However, the direct consideration of the desired endpoint during treatment planning optimisation appears to be the most logical approach for clinical implementation of personalised medicine in prostate radiotherapy. It is expected that the accumulation of PCa radiobiology information and their associated uncertainties will lead to the realisation of patient-specific, biologically-optimised radiotherapy as the standard of care.
